# Influence of Platelet-Rich and Platelet-Poor Plasma on Endogenous Mechanisms of Skeletal Muscle Repair/Regeneration

**DOI:** 10.3390/ijms20030683

**Published:** 2019-02-05

**Authors:** Flaminia Chellini, Alessia Tani, Sandra Zecchi-Orlandini, Chiara Sassoli

**Affiliations:** Department of Experimental and Clinical Medicine—Section of Anatomy and Histology, University of Florence, Largo Brambilla 3, 50134 Florence, Italy; flaminia.chellini@unifi.it (F.C.); alessia.tani@unifi.it (A.T.); sandra.zecchi@unifi.it (S.Z.-O.)

**Keywords:** fibrosis, myoblasts, myofibroblasts, myogenesis, Platelet-Rich Plasma (PRP), Platelet-Poor Plasma (PPP), satellite cells, skeletal muscle regeneration, stem cell niche, regenerative medicine

## Abstract

The morpho-functional recovery of injured skeletal muscle still represents an unmet need. None of the therapeutic options so far adopted have proved to be resolutive. A current scientific challenge remains the identification of effective strategies improving the endogenous skeletal muscle regenerative program. Indeed, skeletal muscle tissue possesses an intrinsic remarkable regenerative capacity in response to injury, mainly thanks to the activity of a population of resident muscle progenitors called satellite cells, largely influenced by the dynamic interplay established with different molecular and cellular components of the surrounding niche/microenvironment. Other myogenic non-satellite cells, residing within muscle or recruited via circulation may contribute to post-natal muscle regeneration. Unfortunately, in the case of extended damage the tissue repair may become aberrant, giving rise to a maladaptive fibrotic scar or adipose tissue infiltration, mainly due to dysregulated activity of different muscle interstitial cells. In this context, plasma preparations, including Platelet-Rich Plasma (PRP) and more recently Platelet-Poor Plasma (PPP), have shown advantages and promising therapeutic perspectives. This review focuses on the contribution of these blood-derived products on repair/regeneration of damaged skeletal muscle, paying particular attention to the potential cellular targets and molecular mechanisms through which these products may exert their beneficial effects.

## 1. Introduction 

Skeletal muscle can be considered as the largest organ of the human body, accounting for 40–45% of the total body mass, responsible for generating forces that guarantee breathing and movement. In addition, it represents an important metabolic and endocrine organ [1,2]. The incidence of skeletal muscle injuries as a consequence of trauma (e.g., sport injuries), inherited genetic diseases (e.g., muscular dystrophies), pathology (cancer and endocrinological disorders) or systemic conditions such as aging is very high worldwide, thus representing a serious socio-economic concern with relevant Health Care System costs [2,3]. Indeed skeletal muscle damage is the most common cause of severe long-term pain and physical disability, restricting patient daily living activities and imposing lost working days. 

It is well known that skeletal muscle tissue possesses an intrinsic remarkable regenerative potential, which, however, becomes compromised in the case of severe and extended damage. In particular, it has been reported that skeletal muscle tissue is able to compensate for up to 20% of muscle mass loss but beyond this threshold the restoration of the native muscle tissue structure and function cannot be achieved [2].

Traditional therapeutic options for the treatment of damaged muscles include RICE (Rest, Ice, Compression and Elevation) treatment, rehabilitation therapies, corticosteroid administration and, in the worst conditions, even reconstructive surgical intervention. The progress of scientific research, that allowed a deeper understanding of the cellular and molecular mechanisms driving skeletal muscle tissue repair/regeneration, together with the advances of regenerative medicine and biotechnology, pushed the development and application of alternative and innovative strategies to promote or improve muscle repair/regeneration, such as cell-based therapy with different kinds of stem cells [4,5,6,7,8,9,10], tissue engineering [11,12] and Low Level Laser Therapy (LLLT—more recently termed Photobiomodulation) [13,14,15].

However, none of the therapeutic options so far adopted has proved to be resolutive and satisfactory; in addition, the most innovative therapeutic strategies, despite the encouraging elicited outcomes, still bear several criticisms hindering their clinical application for regenerative purposes. For more detailed information readers are referred to recent reviews [2,9,10] focusing on this topic, which is beyond the aim of this review. 

Based on these considerations, the development of new effective treatments for skeletal muscle injury represents a priority and an urgent need. A current scientific challenge remains the identification of appropriate factors capable of limiting muscle degeneration and/or potentiating the endogenous muscle regenerative program. In this context, plasma preparations, including Platelet-Rich Plasma (PRP) and more recently Platelet-Poor Plasma (PPP), have shown several advantages and promising therapeutic perspectives. 

This review, besides giving an updated description of the main cell types driving skeletal muscle repair/regeneration, focuses on the contribution of PRP and PPP on repair/regeneration of damaged skeletal muscle, paying particular attention to the cellular and molecular mechanisms through which these blood-derived products may exert their beneficial effects.

## 2. Adult Skeletal Muscle Repair and Regeneration: Role of Satellite and Non-Satellite Cells

It has been clearly proven that adult skeletal muscle tissue possesses a remarkable ability to regenerate in response to focal injuries. This is mainly thanks to the activity of a small population of resident mononucleated myogenic precursors, called satellite cells, whose name depends on the unique anatomical location at the periphery of a skeletal myofiber, beneath the surrounding basal lamina in intimate association with the myofiber sarcolemma, and in close proximity to capillaries and neuromuscular junction [16,17,18,19,20]. In healthy skeletal muscles, satellite cells are mitotically quiescent and transcriptionally inactive; in this dormant state they express the paired box transcription factor Pax7, necessary for their survival and function, and the myogenic transcription factor Myf5 (~90% of quiescent satellite cells) but not the myogenic regulatory factors, namely MyoD or myogenin [21,22]. In injured muscles, in response to signals coming from damaged myofibers and infiltrating inflammatory cells (neutrophils and macrophages), satellite cells become activated, giving rise to a progeny of proliferating Pax7, Ki67, Myf5 and MyoD positive adult myoblasts which subsequently, down-regulating Pax7 and expressing myogenin and MRF4/Myf6, differentiate into skeletal myocytes that finally either fuse with each other, forming nascent syncytial contractile myofibers, and fuse with injured myofibers, thus repairing the damage [21]. There is evidence indicating that a small percentage of satellite cells are *true* stem cells, capable of self-renewal, thereby ensuring the replenishment of the basal pool of resident satellite cells that are recruitable in the case of muscle re-injury [16,23,24].

The behavior and the fate of satellite cells are largely influenced by the dynamic interplay established with components of the surrounding microenvironment, which changes under homeostatic conditions (*quiescent state*) and during regeneration (*activated state*) [25]. This microenvironment includes the so called “immediate satellite cell niche” and the microenvironment beyond the immediate niche [16]. The immediate satellite niche represents the microenvironment where the satellite cells reside. It consists of several signaling molecules diffusing between the satellite cell and the myofiber, different extracellular matrix (ECM) components, satellite cell and myofiber surface-associated receptors for mediating cell-to-cell or cell-to-ECM interactions or binding different regulatory factors, and receptors present in the basal lamina to sequester inactive growth factor precursors secreted by either satellite cells and myofibers, serving as a local reservoir to be rapidly activated during muscle injury [16,26,27]. The microenvironment beyond this niche comprises the local milieu and the systemic milieu. The local milieu may be identified by the muscle fascicle wrapped by perimysium, consisting of other myofibers surrounded and connected to each other by endomysium sheath, a heterogeneous population of interstitial cells in the stroma between the myofibers [28,29,30], blood capillaries together with their secretable factors and associated pericytes and mesoangioblasts [31,32] and motor neuron endings. The main interstitial cells types are represented by fibroblasts, mesenchymal stem/stromal cells (MSCs) including fibro-adipogenic progenitors -FAPs- [33], telocytes (CD34+/vimentin+ stromal cells with a small cell body and distinctive extremely long, thin and moniliform cytoplasmic extensions called telopodes alternating slender segments—podomeres- with dilatations-podoms) [34], Abcg2+ side population (SP) [35], skeletal muscle-derived CD34+/45− (Sk-34) myogenic endothelial progenitors [36], interstitial stem cells positive for stress mediator PW1 expression and negative for Pax7 expression termed PICs [37], integrin β4 interstitial cells.

The systemic milieu includes the entire muscle belly along with bones and surrounding skeletal muscles, the immune cells and circulating growth factors, interleukins/chemokines and hormones.

The dynamic and collaborative interaction between satellite cells and the different cell types of the surrounding microenvironment, becomes crucial for a proper execution of the essential events of repair/regeneration process. Indeed, in contrast to the quiescent conditions, in the activated ones, many cells are found close to satellite cells exerting a supportive role for their functionality. On the other hand, this cell interaction is bidirectional since satellite cells can influence the behavior of interacting cells [20,38]. 

The cells supporting satellite cell-mediated regeneration in the activated niche may include several cell types.

*Pro-inflammatory phagocytic macrophages (M1) and anti-inflammatory pro-regenerative macrophages (M2)* have been demonstrated to promote proliferation and differentiation of myogenic precursors respectively, via both paracrine and juxtacrine signaling [39,40,41,42]. The ability of macrophages to rescue myoblasts and myotubes from apoptosis has also been demonstrated [43].

Fibroblasts-myofibroblasts and FAPs are the major contributors to the deposition and remodeling of the transitional ECM after a muscle lesion, required to rapidly restore tissue integrity [44]; on the other hand the capability of fibroblasts to promote myoblast proliferation and differentiation and to enhance satellite cell renewal as well as pro-myogenic function of FAPs has been documented [38,45,46,47,48,49].

*Telocytes* have been supposed to play a “nursing” role in satellite cell-mediated regeneration. By means of their telopodes they connect with each other via homocellular junctions, or with neighboring cells including satellite cells via heterocellular ones, thus forming a three-dimensional network in the interstitium: telocytes might act as a guidance stromal scaffold able to carry signals over long distances, driving satellite cell proliferation, migration and differentiation after their recruitment [34]. In addition, telocytes may modulate satellite cell function in a paracrine manner by the release of extracellular vesicles containing myogenic factors (e.g., Vascular Endothelial Growth Factor, VEGF, or microRNAs) [4,34,50,51].

*Capillary endothelial cells* secrete different paracrine factors strongly stimulating growth of myogenic progenitors and/or protecting them from apoptosis [19,52,53], whereas *periendothelial cells* including *pericytes* are crucial for the re-entry of satellite cells into quiescence at the end of the regeneration process and myofiber growth [54,55]. 

In addition, *motor neurons and Schwann cells* secreting neurotrophic factors including Insulin Growth Factor (IGF)-1, Nerve Growth Factor (NGF), Brain-Derived Growth Factor (BDNF) and Ciliary Neurotrophic Factor (CNTF) may contribute to the modulation of satellite cell/myoblast viability, proliferation and fusion [16,20,29,56,57].

Furthermore, in regulating satellite cell quiescence, activation, proliferation and differentiation an essential role is played by ECM factors (both of basal lamina and of interstitial matrix) including specific ligands, soluble factors sequestered within the matrix, as well as by the mechanical properties of ECM itself as extensively discussed in the review by Thomas and co-workers [27].

Many works have demonstrated that, in addition to satellite cells, other cell types residing within muscle or recruited via circulation may contribute to muscle regeneration thanks to their inducible myogenic potential [58]. These so-called myogenic non-satellite cells include: the interstitial Abcg2+SP [35,59,60,61], skeletal muscle-derived CD34+/45− (Sk-34) cells (likely a subpopulation of SP with more pronounced myogenic potential) [36], PICs [37], mesoangioblasts and pericytes [31,62,63,64], integrin β4 interstitial cells, CD133+ human skeletal muscle derived and blood- derived stem cells [65,66,67]. However, if these cells represent an independent source of muscle progenitors undergoing unconventional myogenic differentiation or if they give rise to satellite cells, remains to be elucidated. Moreover, also the molecular mechanisms guiding the lineage switch of these muscle interstitial or circulating cells in the regenerating environment are still unclear [28,29]. Based on all of this evidence, it appears clear that, for an effective restoration of muscle structure and function, collaborative and temporally coordinated juxtacrine and paracrine interactions among many myogenic and non-myogenic cells, are required. 

Unfortunately, in case of severe and extended damage, with an intense and persisting inflammatory reaction or in disease settings, the muscle repair may become aberrant, occurring with a maladaptive fibrotic scar or adipose tissue infiltration, or even with heterotopic ossification, mainly as a consequence of dysregulated activity and number of fibroblasts and mesenchymal progenitors [3,33,49,68,69,70], which hamper the muscle regenerative response. Moreover, a critical event that must be considered for the achievement of a regenerating functional muscle tissue after injury is the re-establishment of neuromuscular junctions for the new myofibers, which is mandatory to prevent muscle wasting [71].

On the basis of these considerations, improving the functionality of satellite and non-satellite myogenic cells either directly or by acting on their microenvironment (e.g., modulating the inflammatory response or MSC functionality thus limiting fibrosis or muscle fatty deposition) as well as nerve regeneration, could represent the final goal of effective therapeutic strategies for efficient muscle regeneration.

## 3. Plasma Preparations: Platelet-Rich Plasma (PRP) and Platelet-Poor Plasma (PPP) 

### 3.1. Definition and Biological Properties

PRP and PPP can be defined as a plasma fraction with a concentration of platelets respectively above and below baseline levels in whole blood. Unfortunately, so far, no univocal guidelines are available for these plasma preparations and protocols show a high variability among authors, thus leading to plasma fractions differing in terms of concentration of blood cells (platelets and leukocytes) and content and type of cytokines and growth factors. For a detailed and updated overview of the different methods of plasma fraction collection, of the commercially available PRP systems and of PRP classification, the readers are referred to several excellent reviews [72,73,74,75]. In any case, the rationale for using plasma fractions, in particular PRP, for regenerative purposes in different areas of medicine [76,77,78,79,80,81] including musculoskeletal and sport medicine [82,83,84,85], relies on the fact that they represent a cost-effective reservoir of numerous biologically active molecules, holding a strong potential for improving tissue healing and regeneration [86,87,88,89]. Moreover, the prompt availability from whole blood of patients and thus the autologous source of these blood products, posing no risks of disease transmission or immunogenic reactions [90,91], as well as the ease of administration, represent additional advantages for their clinical use. Furthermore, recent findings demonstrating the safety and favorable outcomes of the application of allogenic PRP to treat musculoskeletal conditions have opened new perspectives for off-the-shelf PRP therapy for all patients for whom the use of autologous PRP would not be recommended [92,93].

### 3.2. Contribution of PRP to Skeletal Muscle Repair/Regeneration

Some studies have reported positive outcomes after administration of PRP in patients with injured skeletal muscles, without negative side effects. Indeed, patients/athletes with acute muscle strains after PRP intralesional injections combined with a rehabilitation treatment, exhibited an earlier “return to play”, faster pain relief without a significant increase of the re-injury risk in short and long term, when compared to patients undergoing a rehabilitation program only [94,95,96,97,98]. Improvement of inflammatory state, reduction of fibrotic scar size and parenchymal recovery was also demonstrated in PRP-treated muscle lesions [94,99,100,101]. However, despite the encouraging findings, these studies do not reach sufficient statistical significance to support the adoption of PRP therapy for skeletal muscle injury in clinical routine, as recently extensively discussed [83,84,102,103,104]. Therefore, further human studies, to ascertain and validate the effective therapeutic benefits of PRP for skeletal muscle regenerative purpose, are strongly required. 

On the other hand, a large body of experimental evidence supporting the contribution of PRP to the morpho-functional recovery of damaged skeletal muscles comes from studies carried out in animal models. Although these studies cannot be directly validated or extrapolated to human species, they do provide a robust and instructive scientific background to design and perform clinical investigations.

In particular, PRP injections into skeletal muscles of rats or mice subjected to different traumatic injuries (incision, laceration, contusion or lengthening/eccentric contractions) or to cardiotoxin injection or into ischemic muscles, have been demonstrated to contribute to the muscle healing process: (i) by modulating the inflammatory response including the increase in M2 macrophage cell recruitment to the injury site and function [105,106,107,108]; (ii) by generating a myogenic response, as evaluated by satellite cell activation, increase of the expression of different myogenic regulatory factors, modulation of the expression of muscle specific microRNAs and activation of myogenic signaling pathways leading to myofiber formation [105,106,109,110,111,112,113]; (iii) by attenuating the impairment of myocytes mitochondrial function determined by muscle damage and improving their endogenous antioxidant defense system [114]; and (iv) by protecting cells from apoptosis [108,111].

In addition, the reduction of type-I collagen deposition and scar formation (fibrosis) [106,110,112,113,115,116,117,118] the enhancement of angiogenesis [106,110,112,116,117] and a faster functional recovery [108,109,113,118,119] have been also observed after PRP application on damaged muscles. Takase and co-workers [120] recently demonstrated also the ability of PRP to prevent fatty degenerative changes of rotator cuff muscles in a rat rotator cuff tear model, when administered into subacromial space. 

### 3.3. Impact of PRP on Satellite Cells and on Myogenic and Non-Myogenic Interstitial Cells 

The cellular and molecular mechanisms that could mediate the beneficial pro-regenerative and anti-fibrotic effects of PRP-derived growth factors on muscle tissue healing have been investigated in a growing number of in vitro studies.

Satellite cells may represent a direct target of PRP action. Indeed, the capability of PRP to positively influence the behavior of primary myoblasts—human skeletal myoblasts [116,121], human pre-plated muscle derived-progenitor cells [122], rabbit myogenic progenitor cells [123], rat intrinsic skeletal muscle cells [124]—or satellite cell-derived myoblast line namely murine C2C12 myoblasts [120,121,125,126] and human CD56+ myoblasts [127] by promoting their activation and proliferation [116,120,121,122,123,124,125,126,127] and protecting them from apoptosis [116] has been demonstrated.

Among the different growth factors within PRP, Platelet Derived Growth Factor (PDGF) [122], has been identified as a key factor mediating PRP-induced mitogenic response, whereas the involvement of others—demonstrated to be contained in PRP [86,87,88,89]—has been proposed on the basis of previous studies investigating their effects on myoblast cell line or primary muscle stem cells, such as VEGF [4,128], Hepatocyte Growth Factor (HGF) [129] or IGF-1 [130]. The involvement of PDGF and VEGF in mediating the PRP effects may be also presumed on the basis of the recent study of Scully and co-workers [131] showing that platelet releasate is capable to drive C2C12 myoblast proliferation and terminal differentiation, as well as the commitment to differentiation of myofiber-derived stem cells, at least in part via PDGF and VEGF signaling pathways.

However, it must be pointed out that the effects of the combination of different growth factors (such as PRP) may be completely different from the ones elicited by the single growth factors, based on the proved antagonistic or synergistic cross-talk between diverse growth factor-mediated signaling. 

In addition, primary cultured skeletal muscle cells treated with PRP have also shown an increased motility/migratory ability associated with an up-regulation of different focal adhesion proteins and F-actin cytoskeleton remodeling [132]; these findings are of particular interest given that migration of satellite cells and of satellite derived-myoblasts is a crucial process in muscle regeneration by which the cells reach the injured site.

In line with these findings, our research group has recently shown that PRP used as single treatment, positively influenced C2C12 myoblast viability and proliferation in the same manner of standard culture media containing animal sera, by promoting the activation of AKT-mediated signaling, as well as the activation of cultured murine satellite cells isolated from single skeletal muscle fibers [133]. 

In this paper, we also demonstrated that PRP treatment induced C2C12 myoblasts to enter and progress into the myogenic program by stimulating MyoD, myogenin, α-sarcomeric actin expression as standard differentiation culture media containing animal sera did, accordingly to a previous study [127]. The pro-myogenic effect of PRP was also recently demonstrated by the study of McClure and co-workers [126] where C2C12 myoblasts cultured on PRP embedded ECM mimicking scaffold, exhibited a PRP-dose dependent increase in myogenin and myosin heavy chain protein expression, mediated by ERK1/2 signaling activation. In addition, our study reported that PRP promoted also the C2C12 myoblast expression of matrix metalloprotease (MMP)-2 [133] whose function has been reported to be required for satellite cell activation [134,135,136], for basal lamina degradation and, at the elongation stage of the myogenic differentiation process, for completing the successive myoblast cell migration and fusion [6,27,137,138] thus supporting the pro-myogenic effect of PRP. These latter results concerning differentiation seem to question findings in the literature showing a reduction or even inhibition of myogenic differentiation of myoblasts cultured with PRP [120,121,125]. These contradictory PRP-elicited biological responses may be attributed to different experimental settings and more likely to the heterogeneity of PRP preparation techniques and formulations used, which may contain interplaying pro-myogenic growth factors—such as IGF-1 [130], HGF [129] and β-Fibroblast Growth Factor (FGF) [125,139]—and anti-myogenic ones—such as Transforming Growth Factor (TGF)-β [140]—in different concentrations and proportions. In addition, the different PRP dosages used may account for the discrepancy in the literature on whether PRP hampers or improves differentiation, when considering the dose-dependence of some myogenic cell responses as well as the timing of PRP application [116,121,125,126,132,133].

Another very interesting finding of our recent work [133] is the ability of PRP to satisfactorily support and stimulate in vitro viability, survival and proliferation of MSCs. Taking into consideration the close morpho-functional relationship between satellite and the interstitial stromal cells, in particular the reported supportive juxtacrine and paracrine role of different stromal cell types for satellite cells, our results may suggest that the muscle resident stromal cells could also benefit from the treatment with PRP. In other words, PRP might indirectly promote satellite cell-mediated regeneration by potentiating the nursing role of interstitial MSCs.

PRP may impact indirectly on satellite cells also by favoring the appearance and functionality of pro-regenerative macrophage phenotype (M2) within the healing niche on the basis of recent in vitro studies, evaluating the effects of different PRP preparations on human monocyte-derived cells [141,142] and also being consistent with the findings of in vivo research [106].

On the other hand, PRP could also affect the myogenic potential of the so-called non-satellite myogenic cells. In this line, Li and co-workers [122] have reported that pericytes, isolated from post-mortem human skeletal muscle biopsies, exhibited an enhanced proliferative ability when cultured with PRP as compared to standard culture media while maintaining their in vitro myogenic differentiation capability and in vivo myogenic potential.

**Figure 1:**
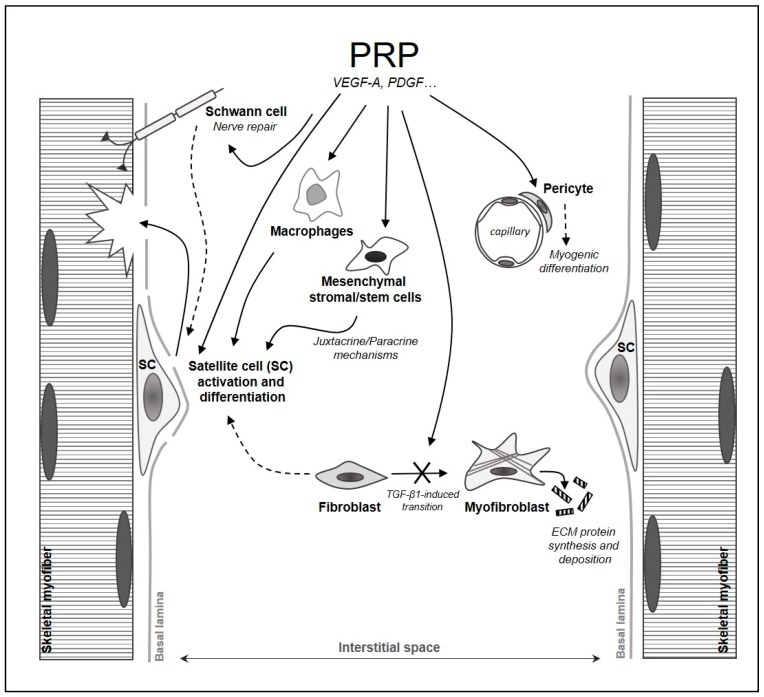
Schematic drawing representing the potential cell targets of PRP during skeletal muscle repair/regeneration, which may mediate its beneficial effects.

Interestingly, we have recently demonstrated the capability of PRP to prevent the transition of fibroblasts into myofibroblasts, the main drivers of tissue scarring [143] via VEGF-A/VEGF-A Receptor-1-mediated inhibition of TGF-β1/Smad3 signaling [144]. These findings, in accordance with other studies [145,146], may contribute to the identification of the cellular and molecular mechanisms responsible for the observed reduction in the fibrotic response achieved after injections of PRP in damaged skeletal muscles [106,110,112,113,115,116,117,118]. Such reduction is necessary for the recreation of a more hospitable and conductive microenvironment for muscle progenitor functionality and for axonal growth/regeneration and muscle re-innervation [147] and eventually for a complete tissue morpho-functional recovery. On the other hand, the ability of different PRP-derived factors to positively influence Schwann cell function in vitro and to assist peripheral nerve repair/regeneration in vivo has been documented [148,149,150,151], thus allowing us to include Schwann cells in the list of the potential cell targets of PRP in the skeletal muscle tissue during tissue repair/regeneration (Figure 1) and to suggest that promotion of muscle re-innervation may represent an additional benefit exerted by PRP for damaged muscles.

### 3.4. Impact of PPP on Skeletal Myogenesis 

PPP, long regarded as the waste product of PRP and used a sham treatment [109], has recently been demonstrated to exert a beneficial effect on myogenesis. In particular, Miroshnychenko and co-workers [121] showed that PPP, differently from PRP, reduced the proliferation rate of human primary skeletal muscle myoblasts but led to a significant induction of differentiation of these cells into the myogenic pathway and myotube formation. The same cell responses were elicited by a modified preparation of PRP, by means of a second spin to remove platelets, suggesting that the beneficial effects of PRP on myogenesis can be mostly due to plasma per se. Indeed, although PPP by its definition contains a very low concentration of platelets and therefore smaller quantities of growth factors, it still represents a reservoir of bioactive molecules (such as PDGF, IGF-1) [87], which may be responsible for the observed pro-myogenic effects on myoblasts. This research is in line with previous studies showing the effectiveness of PPP in evoking biological responses from different cell types involved in the healing process of several tissues, namely fibroblast migration and ECM remodeling [152,153], periodontal ligament stem cell differentiation towards osteoblastic phenotype [154], tenocyte proliferation and collagen production [155], endothelial cell differentiation towards angiogenic cells [156] and macrophage anti-inflammatory activity [157]. Miroshnychenko and co-workers [121] conclude that PPP and platelet deprived-PRP may be more appropriate to promote skeletal muscle regeneration than the traditionally formulated PRP, probably containing a greater quantity of platelet- derived factors detrimental for myoblast differentiation such as TGFβ-1. However, this laboratory evidence does not seem to have received a large consensus from the current literature [103,109].

Therefore, although PPP may hold promise for skeletal muscle injuries, further investigations to assess the exact growth factors contained in this kind of plasma formulation and especially to validate the impact of PPP on myogenic precursors, as well as to disclose its role on skeletal muscle tissue regeneration in vivo are absolutely required. Moreover, to date, no clinical studies evaluating the effect of PPP on skeletal muscle have been conducted.

## 4. Conclusions and Further Directions

Plasma preparations and especially PRP have been demonstrated to hold a strong therapeutic potential for the healing of injured skeletal muscle tissue due to their ability to potentiate the endogenous mechanisms of tissue repair/regeneration while contributing to limit the aberrant responses such as fibrosis. Nevertheless, despite these encouraging outcomes, evidence from animal studies and even more from human ones, are not still sufficient to attain the effective clinical translation of these blood products for skeletal regenerative purpose. Therefore, further investigations are necessary to validate the regenerative potential of these blood derivates and ultimately drive medical decisions. On the other hand, it must be considered that some human and animal studies have reported limited effectiveness or inefficacy of a PRP therapy for damaged skeletal muscle in terms of tissue regeneration and recovery of functionality, or even an exacerbation of the fibrotic response [158,159,160,161,162,163,164,165,166,167,168]. The main reason for these conflicting results certainly could be due to individual-based variations and muscle lesion type and severity but is most likely due to the great heterogeneity of the injected available products and application timing. Therefore, standardization of PRP preparation techniques as well as of application protocols—considering the cascade of events through which skeletal muscle tissue repair/regeneration progresses—is a priority in order to perform meaningful comparative analyses, to enable reproducibility and reach reliable conclusions. Moreover, a full characterization of plasma preparations is also necessary, evaluating the quantity and the type of the contained bioactive factors, as well as a deep investigation of their effects on the main local cell types involved in the skeletal muscle tissue regeneration. This may lead to novel and optimized plasma formulations for muscle regenerative purposes, based on the selection of factors capable, for instance, of exerting a pro-regenerative and anti-fibrotic action on skeletal muscle tissue.
